# Holding space open: Wor(l)ding the ruins of Gaza

**DOI:** 10.1177/23996544261466065

**Published:** 2026-06-29

**Authors:** Mikko Joronen, Khalid Dader

**Affiliations:** 1 7840 Geographies of Coloniality and Everyday Violence Research Group (GOCEP), Administrative Studies, Tampere University, Tampere, Finland; 2 Environmental Policy and Regional Studies, Tampere University, Tampere, Finland

**Keywords:** writing, Gaza, embodiment, erasure, annihilation of space

## Abstract

Witnessing the erasure of spaces and landscapes in Gaza reveals as much the ethical and ontological necessity to narrate as it does the limits of writing. In this commentary on Lubna Abu Sitta’s intervention, we grasp this tension by thinking with her how embodied writing can narrate what seems impossible for words to capture. Abu Sitta’s writing in-place and through lived ruination voices affects, attachments, vulnerabilities, losses, and exposures that circumnavigate bodies, while importantly holding space open for memory and life in a way that resist immense spatial erasure. This urges us to think embodied displacement geographically, bringing attention to ways of inhabiting the ruins and destruction with makeshift infrastructures, memories, and narrations of survival. These ways and the losses they carry might not lend themselves easily to academic narration, but they are nevertheless carved into bodies, memories, affects, atmospheres, materialities and landscapes—to spaces and bodies that are worlding the impossible in the ruins of Gaza.

During the last 3 years, writing has been at times the only means through which solace can be mustered in a way that maintains sanity. Writing has offered a way to articulate and share the embodied connections to Gaza, while also serving as a documentation and witness that, in the academic spaces, can also help (un)learning from, drawing on, and thinking anew—in ways that flag the inadequacy of current academic vernaculars while inviting thinking to grasp Gaza with proper ethical language. Writing remains a powerful tool of resistance and solidarity too, while also affording testimonies that shake the propagandist apologetics surrounding Gaza as the intervention of Lubna Abu Sitta we respond to herein shows in clarity. And yet, it is in places like Gaza that also the limits of writing come to the fore in profound ways. As (Salamanca et al., 2024) asked in an intervention published in April 2024, “how does one write in the midst of an ongoing genocide? When the contours of Palestine are being redrawn in blood, and when unconscionable images of starving, injured, and dead children, women, and men have become our daily breakfast?”. Perhaps, they added, “no language, no cartography, no record is capable of tracking, conveying, mapping, archiving the ongoing lived reality of this devastation”—the killing en masse, the persistent fabrication of falsehoods, the complete annihilation of landscapes and spaces of living; all that should make us question whether genocide, “the crime of crimes, bears any explanation at all” (Salamanca et al., 2024).

Indeed, there is a fundamental tension present when writing about the horrors of Gaza, particularly as the events are still unfolding and ongoing. Perhaps on some occasion silence can remain the strongest witness too. In Gaza the limits of scholarly language become evident, but so too comes the need to hear the words of those—like Lubna Abu Sitta—who have lived through, and survived, what the academic language struggles to find proper words to describe. This is what Lubna’s intervention pens down in clarity: the need to write—to narrate and put into words, and affect, what seems impossible to speak of. Abu Sitta (2026) commentary writes the impossible, narrates what seems to escape the words.

The impossible thus remains bound to a call—to the arrival of events so dreadful and monstrous that they grave for a new language and proper ethics of speaking. The events in Gaza demand us to speak, not the least as the events have been widely narrated by those who do not shame to articulate them through familiar colonial, racialised and imperial tropes and alignments, at times through outright lies and hasbara. Even when emancipatory, as Otman and Shalhoub-Kevorkian (2026) write, in a context of Palestinians studying at an Israeli academic institution, the use of coloniser’s language to reveal and challenge their own oppression can play into racialized colonial agendas in ways that are not merely a question of language but also embodied, carving the flesh of the colonized with an emotional toll they bear. Yet, these words of colonisers—of Amalek, water cuts, starvation, erasure, expulsion, murder, ecocide, spaciocide, and more (Ahmed et al., 2024; HRW 2024; Joseph 2025; Omer 2026)—need be brought to light through their material and embodied effects. To call for justice and to show the long settler ambition to colonize Palestine, they need to be let to speak through the bodies and sites exposed to their colonial violence. Such language of exposure emerges from bodily vulnerability and the layers of lived landscapes; rather than repeating the claims of those captivated by their own destructive abilities, it gives words to what connects life and makes us dependent on the care of others, thereby revealing the sites, materialities and ecologies of living from which those words emerge.

Naturally, silences too can speak in many ways. They may be the silences of those lost—murdered—allowing silence itself to speak and to make space for lost lives and worlds. But silences can also lurk at the shadows of normalizing the devastation, as is well-exemplified by the long silence on Gaza by many Western mainstream media outlets—all despite the fact that scholars, human right organisations, and the UN had early on warned of the crime genocide, while the destruction was effectively broadcasted live and widely documented by those living amid the inferno of burning Gaza (Agha et al., 2024; Bearing Witness, n d; Genocide.live, n d). The ‘impossible’ thus calls for a proper narration—ethical, critical, material, embodied—as much as silences need to be pointed out when they work to diminish one of the most intensive phases in more than hundred years of settler colonial violence in Palestine (see Khalidi 2020; Sabbagh-Khoury 2023; Said 1979).

There is much to grasp on Abu Sitta’s rich commentary speaking with direct, personal, affective, and spatial tone on the matters mentioned above. Her words describe the erased world(s), landscapes and everyday spaces, while also bringing alive the altered realities of living amid annihilated spaces and their unfamiliar familiarity (Järvi 2024). There remains a geographical undertone in Abu Sitta’s commentary too, the genocide being articulated firsthand by someone who not only has a background in studying geography but writes in ways that divulge key geographic realities while picturing the landscapes of living with and beyond the settler colonial erasure. Writing in-place and about the materialities present—holding space open for memories while witnessing vast ongoing spatial transformation—speaks to, engages with, and importantly urges us to think geographies juxtaposed with displacement and its spatial intricacies, materialities, and recalibrations with emotion as much as through embodiment.

Let us shortly rewind and zoom out to the rapidly shifting geographies of Gaza. The first 2 weeks of the genocide in 2023—not presented here as a start, but as a moment of intensifying the colonization of Palestine through accelerating and openly devastating genocidal violence—saw mass destruction translate into large-scale displacement from the bordering areas of colonial enclave. This preceded various ethnic cleansing-oriented displacement campaigns that, over the two following years, moved in divergent and at times contradictory directions—simultaneously performing propagandist military foils and engineering spaces of entrapment, annihilation and colonisation. Today, it remains strikingly clear how Israeli displacement calls to ‘safety zones’ functioned to expel and entrap; during the first months, for instance, Gazans were forced to move into Rafah, only to later reduce Rafah to rubble (from May 2024 onwards), while pushing the population under fire to the middle area in Deir al Balah (Amnesty International, 2025; Forensic Architecture 2026). Once the starvation phase started to take more drastic shape due to Israel’s prevention and continuous hampering of humanitarian aid to enclosed Gaza, entrapments also localized, turning into hunger games-like struggle for food and clean water (Anonymous, 2024; Dader 2025). Israeli- and US-run food distribution sites time and a time again turned into killing fields for the masses trying to find food for their families to merely survive. The entrapment logic was also tied to land grabbing—materializing colonial logics of spatial erasure and clearing (see Joronen, 2025; Masalha, 2023; Toscano and Bhandar 2025)—when entire villages and neighborhoods were destroyed and bulldozed after taken under Israeli military control (Forensic Architecture 2026).

In practice, the situation on the ground was reconfigured, first, through the enforced inaccessibility to large sections of the enclave under Israeli military control, and then, through subsequent mass destruction and ‘cleansing’ of these spaces—partly conducted by non-military contractors bulldozing the premises—demarcated as the yellow line buffer zone (Al Jazeera, 2026; Forensic Architecture 2026). Bantustans, reservations, enclaves, buffer zones, yellow lines—colonial reworking of the land-population dyad comes with many names (Abdulla, 2016; Brooks and Griffiths 2024; Falah, 2005; Zureik 2016). Second, spatial transformation qua destruction and flattening of urban landscapes forced the Gazan population into makeshift tent “cities” and to resort what is also at the heart of Abu Sitta ‘s intervention: the facilities like universities, schools, and hospitals, which, as we have seen, have been attacked multiple times despite widely known to shelter civilians. Third, Gazans recalibrated and reconfigured spaces through practices we called elsewhere ‘fitful infrastructures’ (Dader and Joronen, 2025), thus inhabiting ruins and other spaces rendered unfit for inhabitation and deprived of dwelling necessities.

In Luban’s intervention, these three spatial aspects are lucidly present. She encapsulates them, perhaps the best, when writing about the ‘green squares’ of the university being “now filled with dust and tents”—”what were once classrooms and places of learning, are now improvised spaces of shelter”. The commentary further highlights the role of writing in restoring these ‘severed spaces’, as Abu Sitta calls them. “The geographical knowledge planted in us by our academic mentors does not fall with the buildings”, she holds, stressing writing not only as a way of documentation and witness but as a tool for resisting erasure—bodily, ontological, epistemological. Abu Sitta’s descriptions of materiality through her writing in-place—as being-there—remains tightly related to restoration of lived spaces. The classroom, the geography studio, the lecture halls, the green, leafy, and spacious landscape of the university, which Israel destroyed, as every other university in Gaza, is present and alive in narrations that capture the university as a place, where the knowledge and the teachers are still visiting “the corridors of [her] memory”. In writing down these memories and lived experiences, along with the material transformations their destruction embodies, Abu Sitta’s text speaks of and against the colonial transformation, the consequences of vast and swift erasure of places—the halls, the corridors, the green spaces, the favorite corner; the parallel world—but also the spaces, the geomorphology of the land present in the field trainings, for instance.

Writing as embodiment, as a way of being-in place, is what we want to conclude with our short commentary on Abu Sitta’s intervention. Writing is a bodily task, as we navigate ideas, arguments, and encounters through our bodies (Probyn 2010), and thus, invoke, ideate, draft, and decide words that are let to speak for what cannot be fully captured with words. To think writing vis-à-vis writing against and beyond the captures of erasure is to voice out the ideas that circumnavigate through the bodies—affects, attachments, vulnerabilities, losses and exposures to unimaginable. Ideas are not simply ‘mundane’ ideas; they are also realities, encounters, and memories repeated in real time—of loss, grief, pain, shock, displacement, exhaustion, scarcities and absences; of erased homes, streets, neighborhoods, cities, daily rhythms, and worlds.

These embodied spaces are also shared, including public spaces as Abu Sitta’s writing on the attack on education facilities, knowledge-production sites, and learning environments in Gaza shows. This underlines how scholasticide is not only directed on academic institutions but to the erasure of lived and embodied spaces of learning (see also Alarabeed, 2025). In our short editorial on scholasticide written early on—which Abu Sitta referred to (Dader et al., 2024)—we wanted to remind that the systematic destruction of Gazan universities, educational spaces and archives of memory is not a one-off event, but part of a long history of attacking education in Palestine with ways that also affect universities and educational spaces elsewhere, including Finland where we are based. The ‘intimate scholasticide’, as Abu Sitta puts it, provides us a further ‘map of loss’, encapsulating well what genocides intend, do, and maintain against the people they aim to erase, not only when peaking in intense and spectacular moments but also when encroaching through decades of slow elastic settler colonial expansion in and erasure of Palestine (Amira 2021; Joronen 2021; Weizman 2007).

The abundance of losses in Gaza might be too much to narrate—too hard to find words for, too many to engage with, too plenty to simply list here—but every single one narrated can offer a way to navigate through the embodied losses, let alone the grief and weight in them. These losses might not lend themselves easily to become subjects of academic thought, but they are nevertheless carved into bodies, memories, affects, atmospheres, materialities and landscapes—to spaces and bodies that are worlding the impossible in the ruins of Gaza.
